# Supplementary material for: A glutaredoxin domain fused to the radical-generating subunit of ribonucleotide reductase (RNR) functions as an efficient RNR reductant
Source: J Biol Chem. 2018 Aug 30;293(41):15889–900. doi: 10.1074/jbc.RA118.004991 (PMC6187632; doi:10.1074/jbc.RA118.004991)
Supplement: Supporting Information [file supp_293_41_15889_v2_index.html]

A glutaredoxin domain fused to the radical-generating subunit of ribonucleotide reductase (RNR) functions as an efficient RNR reductant — Glutaredoxin and ATP-cone fusions to ribonucleotide reductase — Supporting Information 

# A glutaredoxin domain fused to the radical-generating subunit of ribonucleotide reductase (RNR) functions as an efficient RNR reductant

## Supporting Information

- Supporting Tables and Figures
