# Supplementary material for: A glutaredoxin domain fused to the radical-generating subunit of ribonucleotide reductase (RNR) functions as an efficient RNR reductant
Source: J Biol Chem. 2018 Aug 30;293(41):15889–900. doi: 10.1074/jbc.RA118.004991 (PMC6187632; doi:10.1074/jbc.RA118.004991)
Supplement: Supporting Information [file supp_RA118.004991_JBC-2018-004991R1-Supporting_Information_to_be_published_online.pdf]

SUPPORTING INFORMATION

A glutaredoxin domain fused to the radical-generating subunit of ribonucleotide reductase (RNR) functions as an efficient RNR reductant

Inna Rozman Grinberg[1], Daniel Lundin[1], Margareta Sahlin[1], Mikael Crona[1,2], Gustav Berggren[3], Anders Hofer[4], Britt-Marie Sjöberg[1]*

From the [1]Department of Biochemistry and Biophysics, Stockholm University, Sweden; [2]Swedish Orphan Biovitrum AB, Stockholm, Sweden; [3]Department of Chemistry, Uppsala University, Uppsala, Sweden; [4]Department of Medical Biochemistry and Biophysics, Umeå University, Umeå, Sweden.

Table S1. RNR proteins with a fused glutaredoxin domain. Presence of ATP-cone is indicated. RefSeq data from March 16, 2018.

Protein composition	Taxon	RNR class	RNR subclass	RefSeq accnos
ATP-cone: Glutaredoxin:NrdA	Cafeteria roenbergensis virus BV-PW1	NrdA	NrdAe	YP_003970087.1, YP_003970181.1
ATP-cone: Glutaredoxin:NrdA	Chrysochromulina ericina virus	NrdA	NrdAe	YP_009173474.1
ATP-cone: Glutaredoxin:NrdB	Facklamia ignava	NrdB	NrdBi	WP_006702003.1, WP_101954394.1
Glutaredoxin:NrdA	Streptococcus pneumoniae	NrdA	NrdE	WP_078376953.1
Glutaredoxin:NrdA	Cyanophage S-TIM5	NrdA	NrdAi	YP_007006137.1
Glutaredoxin:NrdA	Deftia phage phiW-14	NrdA	NrdAk	YP_003358882.1
Glutaredoxin:NrdB	Allofrancisella guangzhouensis	NrdB	NrdBk	WP_039124745.1
Glutaredoxin:NrdB	Francisella halioticida	NrdB	NrdBk	WP_088772808.1
Glutaredoxin:NrdB	Francisella hispaniensis	NrdB	NrdBk	WP_066046290.1
Glutaredoxin:NrdB	Francisella noatunensis	NrdB	NrdBk	WP_014715631.1
Glutaredoxin:NrdB	Francisella persica	NrdB	NrdBk	WP_064461048.1
Glutaredoxin:NrdB	Francisella philomiragia	NrdB	NrdBk	WP_012280920.1, WP_035735545.1, WP_042523441.1, WP_044526306.1
Glutaredoxin:NrdB	Francisella sp. CA97-1460	NrdB	NrdBk	WP_071664413.1
Glutaredoxin:NrdB	Francisella sp. FSC1006	NrdB	NrdBk	WP_040007580.1
Glutaredoxin:NrdB	Francisella sp. MA067296	NrdB	NrdBk	WP_071629022.1
Glutaredoxin:NrdB	Francisella sp. TX076608	NrdB	NrdBk	WP_071514393.1
Glutaredoxin:NrdB	Francisella sp. TX077308	NrdB	NrdBk	WP_041263735.1
Glutaredoxin:NrdB	Francisella sp. TX077310	NrdB	NrdBk	WP_072712347.1
Glutaredoxin:NrdB	Francisella sp. W12-1067	NrdB	NrdBk	WP_035718892.1
Glutaredoxin:NrdB	Francisella tularensis	NrdB	NrdBk	WP_003015818.1, WP_003018891.1, WP_003023235.1, WP_003026158.1, WP_003033975.1, WP_003036631.1, WP_014548387.1, WP_025329333.1
Glutaredoxin:NrdB	Francisella	NrdB	NrdBk	WP_004287627.1

Glutaredoxin:NrdB	Ectocarpus siliculosus virus 1	NrdB	NrdBe	NP_077613.1
Glutaredoxin:NrdB	Caulobacter virus Karma	NrdB	NrdBk	YP_006989492.1
Glutaredoxin:NrdB	Caulobacter virus Magneto	NrdB	NrdBk	YP_006988790.1
Glutaredoxin:NrdB	Caulobacter virus phiCbK	NrdB	NrdBk	YP_006988007.1
Glutaredoxin:NrdB	Caulobacter virus Rogue	NrdB	NrdBk	YP_006989137.1
Glutaredoxin:NrdB	Caulobacter virus Swift	NrdB	NrdBk	YP_006989840.1
Glutaredoxin:NrdB	Bathycoccus sp. RCC1105 virus BpV1	NrdB	NrdBe	YP_004061574.1
Glutaredoxin:NrdB	Micromonas sp. RCC1109 virus MpV1	NrdB	NrdBe	YP_004062038.1
Glutaredoxin:NrdB	Ostreococcus lucimarinus virus 1	NrdB	NrdBe	YP_004061759.1
Glutaredoxin:NrdB	Ostreococcus lucimarinus virus 2	NrdB	NrdBe	YP_009172647.1
Glutaredoxin:NrdB	Ostreococcus mediterraneus virus 1	NrdB	NrdBe	YP_009172920.1
Glutaredoxin:NrdB	Ostreococcus tauri virus OtV5	NrdB	NrdBe	YP_001648250.2
Glutaredoxin:NrdB	Caulobacter phage CcrColossus	NrdB	NrdBk	YP_006988376.1
Glutaredoxin:NrdB	Micromonas pusilla virus 12T	NrdB	NrdBe	YP_007676142.1
Glutaredoxin:NrdB	Ostreococcus tauri virus 1	NrdB	NrdBe	YP_003212974.1
Glutaredoxin:NrdB	Ostreococcus tauri virus 2	NrdB	NrdBe	YP_004063573.1
Glutaredoxin:NrdB	Pseudoalteromonas phage H101	NrdB	NrdBk	YP_009225573.1
Glutaredoxin:NrdB	Shewanella sp. phage 1/4	NrdB	NrdBg	YP_009100336.1
Glutaredoxin:NrdB	Vibrio phage eugene 12A10	NrdB	NrdBg	YP_009223038.1
Glutaredoxin:NrdB	Vibrio phage helene 12B3	NrdB	NrdBg	YP_007877328.1
Glutaredoxin:NrdB	Vibrio phage ICP1	NrdB	NrdBg	YP_004251147.1
Glutaredoxin:NrdB	Vibrio phage PWH3a-P1	NrdB	NrdBg	YP_007675959.1
Glutaredoxin:NrdB	Vibrio phage qdvp001	NrdB	NrdBk	YP_009222074.1
Glutaredoxin:NrdD	Lachnospiraceae bacterium TWA4	NrdD	NrdDd	WP_082039398.1
Glutaredoxin:NrdJ	Labrenzia aggregata	NrdJ	NrdJm	WP_082444658.1

Table S2. Presence of glutathione biosynthesis and glutathione reductase genes in *Facklamia* spp. A minus sign denotes that no similar protein sequence was identified by Blast.

Gene	*F. ignava*	*F. sourekii*	*F. miroungae*	*F. hominis*	*F.* sp. HMSC062C11	*F. languida*
gshA (*E. coli* K12)	EKB58368 (bifunctional)<br>WP_083857132 (bifunctional)<br>WP_101953697 (bifunctional)<br>PKY90772 (bifunctional)	WP_028119450 (bifunctional)	SDG21584 (only 382 aa)	WP_006908210 (bifunctional)<br>WP_101975023 (bifunctional)<br>PKY93223 (bifunctional)<br>WP_016648844<br>. (bifunctional)<br>EPH07821 (bifunctional)	WP_070608971 (bifunctional)<br>OFL65675 (bifunctional)	WP_006308547 (bifunctional)<br>EHR37891 (bifunctional)
gshB (*E. coli* K12)	-	-	-	-	-	-
gshF (*L. mono-cytogenes*)	EKB58368 (bifunctional)<br>WP_083857132 (bifunctional)<br>WP_101953697 (bifunctional)<br>PKY90772 (bifunctional)	WP_028119450 (bifunctional)	SDG21584 +<br>SDG21622<br>(382 + 348 aa)	WP_006908210 (bifunctional)<br>WP_101975023 (bifunctional)<br>PKY93223 (bifunctional)<br>WP_016648844<br>. (bifunctional)<br>EPH07821 (bifunctional)<br>EKB54699	WP_070608971 (bifunctional)<br>OFL65675 (bifunctional)	WP_006308547 (bifunctional)<br>EHR37891 (bifunctional)
gor	-	WP_028119450 (bifunctional)	-	-	-	-

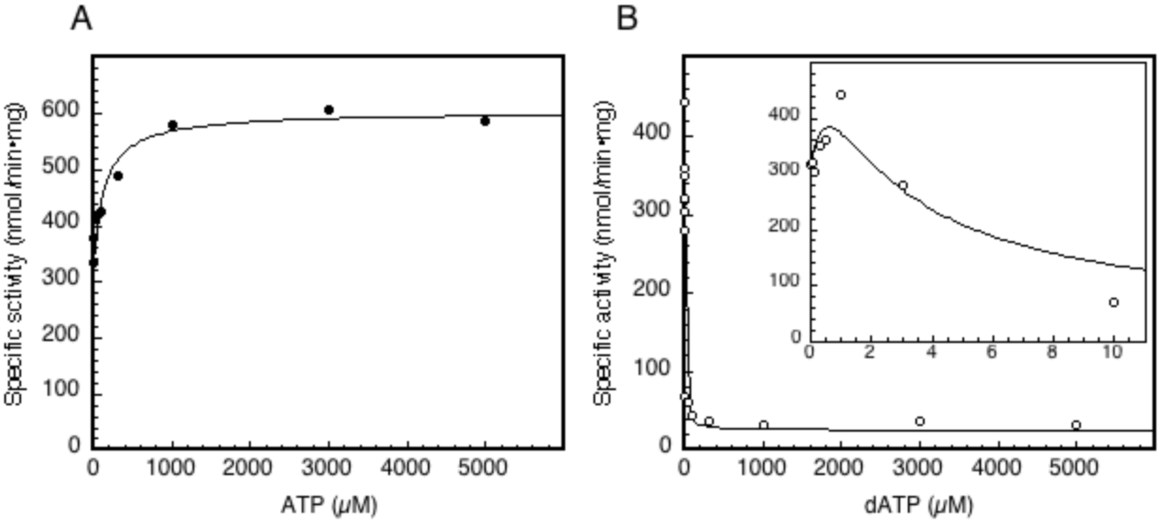

Figure S1. Enzyme activity of *F. ignava* RNR in presence of ATP or dATP. The NrdA protein used was before HIC chromatography and has an intrinsic activity of approximately 300 nmol/min•mg. A) ATP titrations showing an apparent K_L of 150 µM. B) dATP titrations; the *inset* shows that dATP promotes enzyme activity when binding with high affinity to the specificity site in NrdA and inhibits the enzyme activity with binding to the ATP-cone in NrdB at dATP concentrations above 2 µM. Two independent binding sites for dATP were evaluated according to the equation: $v=v_{max1}\times[\text{dATP}]/(K_{L1}+[\text{dATP}])+v_{max2}\times[\text{dATP}]/(K_{L2}+[\text{dATP}])$.

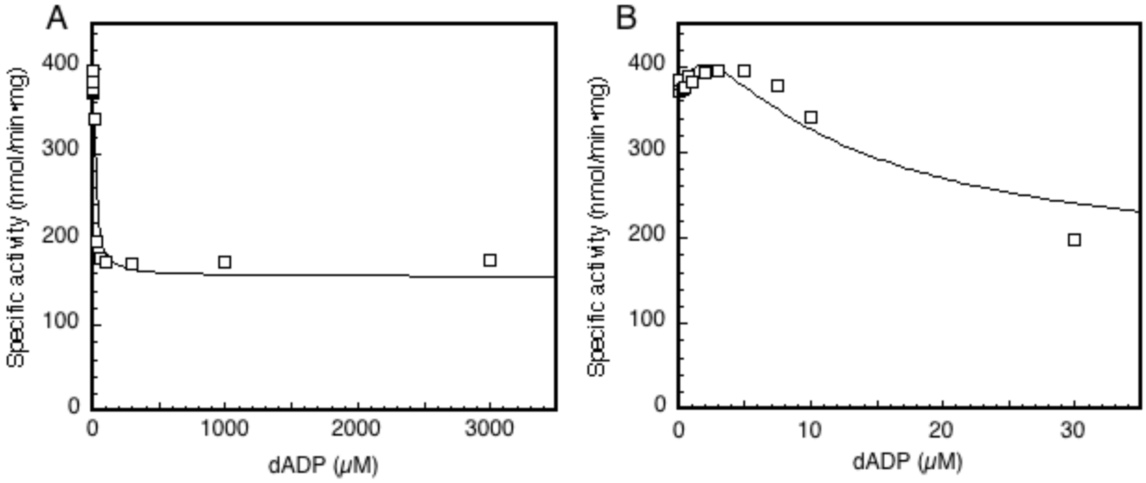

Figure S2. dADP titration of *F. ignava* RNR. The NrdA protein used was before HIC chromatography and has an intrinsic activity of approximately 300 nmol/min•mg. A) Full scale of measurements, and B) blow-up of the lower concentrations of dADP highlighting that enzyme activity is inhibited above 5 µM.

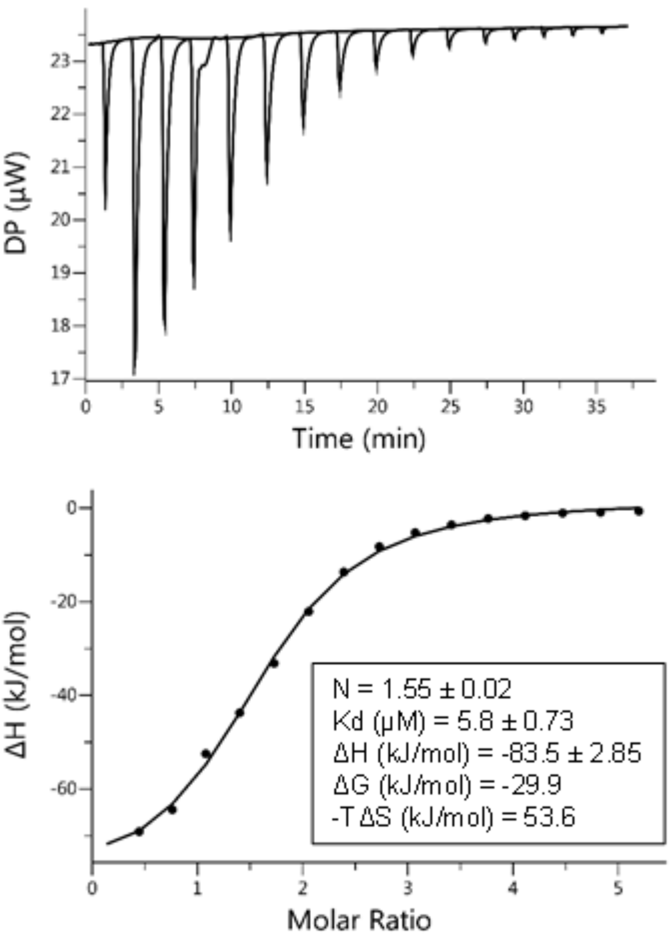

Figure S3. dADP binding to *F. ignava* NrdB. ITC was performed as described in Experimental Procedures in a buffer containing 25 mM Hepes at pH 7.6, 150 mM NaCl, 10% glycerol, 5 mM $Mg(CH_3CO_2)_2$ at 25°C. Cell and syringe contained 30 µM NrdB and 750 µM dADP respectively.

```
FiNrdB MTQVQVYSKKVCPWCTRLKDWMDKNNIDYEEIDIEVHPAREYLHSLGLKTVPQVFIDGVH 61
LbNrdB -----------------------------------------------------------

FiNrdB RPHEPLYSNLLHYIHGTSKIPQHVTKRNGDRVDFDPTKIEIAIQKA-------NNETHE 113
LbNrdB ---------------MSSQEIKKIIKRDYSTAPFVLEKITNAIANAMAALGHGSEQDAK 44
                       :*:  ::: **: . . *   **  ** :*       .::  :

FiNrdB LNDMEINTVTQKVVEKIDIDPIHVEEIQDLVETELMLQSFPRTAKAYILYRAEQTKARQR 173
LbNrdB LISMQVYESLLNNKEQESEYIPTVEQVQDMVEDKLMSSEFHDVAKAYIIYRNKRALERKT 104
       * .*::     :  *: .     **::**:** :** ..*  .*****:** :::  *:

FiNrdB DIFKPRKNLKPYEYPELESYKEAIQHSYWLHTEYNYTSDIQDYKINVEPHERTAIRNAML 233
LbNrdB NIFEKRINLKPYEYPELNEYVAAIRHSYWIHTEFNFTSDIQDFKTGLSEVERSAIKNTML 164
       :**: * **********:.*  **:****:***:*:******:* .:.  **:**:*:**

FiNrdB AISQVEVSVKTFWAKLYDRLPKPELANVGMTFAESEVRHSDAYSHLLELLGLNEIFEEIE 293
LbNrdB AISQIEVAVKTFWGDVHHRLPKPEIAAVGATFAESEVRHHDAYSHLLEILGLNEEFKELK 224
       ****:**:*****..::.******:* ** ********* ********:***** *:*::

FiNrdB QIPALNQRVDYLNSHIKYSQTGSNRDYTISILLFSAFIEHISLFSQFLIIMAFNKQKNLF 353
LbNrdB KKPVIMKRVHYLETSLKHAKSDDDREYTESILLFALFIEHVSLFSQFLIIMAFNKHKNML 284
       : *.: :**.**:: :*::::..:*:** *****: ****:**************:**::

FiNrdB SGISNVIEATSKEEQLHGEFGIDLINTIKAENPDWFDDELKEEIYTLVKQAYESEKDVLD 413
LbNrdB KGISNAVEATSKEEQIHGDFGVDIINIIKKENPEWFDEEHNNLIKEMCLNSFEAESKVVD 344
       .****.:********:**:**:*:** ** ***:***:* :: *  :  :::*:*..*:*

FiNrdB WIYEDGEIDFMPTQTVLEFIKNRLNNSLEAIGLERIFETDLAEVEKTLWFDEEIVSTKHV 473
LbNrdB WIFEKGELDFLPKAVINEFLKNRFNKSLEAIGLEKLFDIDEALLQETEWFDDEIIGTKHG 404
       **:*.**:**:*. .: **:***:*:********::*: * * :::* ***:**:.***

FiNrdB DFFAKRSVNYTKRSQSMTADDLF 496
LbNrdB DFFVKRSINYSKRTQSITSDDLF 427
       ***.***:**:**:**:*:****
```

Figure S4. BLAST alignment of NrdB sequences from *F. ignava* and *L. blandensis*. Highlighted domains are Grx corresponding to pfam00462 (*yellow*) and ATP-cone corresponding to pfam03477 (*green*). The conserved redox-active cysteines in the Grx domain are indicated in red, and the starts of *F. ignava* NrdBΔGrx and NrdBΔ169 are indicated with red arrows. Residues involved in the binding of the two dATP molecules in *L. blandensis* NrdB (1) are indicated in blue for one dATP molecule and red for the other dATP molecule. The ATP-cone domains in *F. ignava* NrdB and *L. blandensis* NrdB have a sequence identity of 28%, and the NrdB cores (covering residues 170-496 in *F. ignava* NrdB and 101- 427 in *L. blandensis* NrdB) have a sequence identity of 61%. Alignments were made using Clustal O at the UniProt server (2).

```
FiNrdA --------------------------------MTAQLKNREYEPFYWLTEDSQTFLERGY 28
LbNrdA MRENTTKLENSTETNVQHTKDNDLLKARRDALQDATEKNTDEPSFDWLTEHSRSFLAAGY 60
                                         *  ** :   * ****.*::**  **

FiNrdA LREGQDAHSRLREIADRAEEILGIDGFSDKFYTYLGRGYYSLSTPVWTNFGSNRGFPISC 88
LbNrdA LSEGVSAEERIREIADRAEEILRMPGFSDKFYKYMGEGYFSLASPVWSNFGKKRGLPISC 120
       * ** .*..*:*********** : *******.*:*.**:**::***:***.:**:****

FiNrdA FGSTPDDNMASILYTAAEIGMMSKYGGGTSGFFGHLRPRGAAITNNGYSSGAVHFMKLFE 148
LbNrdA FGSHIDDDMGNILYTQSEVGMMSKLGGGTSGYFGKIRHRGAAVKNNGYASGAVHIMQLFD 180
       ***  **:*..**** :*:***** ******:**::* ****:.****:*****:*:**:

FiNrdA QMTDTVSQGSSRRGRFAPYLPIDHPDIDEFLEIGVEGNDIQDLNHAVTVPDDWMREMIQG 208
LbNrdA KMVDVVSQGSVRRGRFSPYLPISHPDIKEFLEIGTEGNSIQQLTHGVTVDSTWMQEMIDG 240
       :*.*.***** *****:*****.****.******.***.**:*.*.*** . **:***:*

FiNrdA DREKRATWAKVIQRRVEMGYPYILFTDTINNNKPEWYHDY--PITHSNLCSEIMLPDNDH 266
LbNrdA DTDKREVWAKVLQRRGEMGYPYIFYTDNANNGKPDVYKDKGHDIYASNLCTEIMLPSSDE 300
       * :** .****:*** *******::**. **.**: *:*    *  ****:*****..*.

FiNrdA WSFVCNLSSMNVLHYNEWKDTDAVETMIYFLDAVMSEFIEKLEKLRDSDQFEYREAFHFM 326
LbNrdA WSFVCVLSSINVLHYDKWKNTDAVETMVCFLDAVLTEFIDKLEEYRDSDNRDHRQTFMFM 360
       ***** ***:*****::**:*******: *****::***:***: ****: ::*::* **

FiNrdA ERAYNFAVENRALGLGVLGWHSLLQSNLIAFESEEASYLNEEIHRTIQERAEKASCELAE 386
LbNrdA ERAYNFAKSNRALGLGVLGWHSLLQSKRHAFDSQEAYDLNSEIFREIKQRSYKASEELAE 420
       ******* .*****************:  **:*:**  **.**.* *::*: *** ****

FiNrdA LFGEPLILKGTGRRNTTTMAIAPTTSSAFIIGQVSQSIEPLFSNYYVKDMSKIKTTIRNR 446
LbNrdA KFGEPETLKGYGRRNATLNAIAPTTSSAFILGQVSQGIEPIWSNVYVKDIAKIKTTIKNP 480
        ****  *** ****:*  ***********:*****.***::** ****::******:* 

FiNrdA YLVELLEEKGRNTPDVWESIKNHDGSVLHLDFLSDHEKNVFKTFAEINQYEIINQAAVRQ 506
LbNrdA FLEELFEEKGMNTPEVWRSVRDNDGSVQHLEFLTEQEKDVFKTYAEIDQMAIIYQAANRQ 540
       :* **:**** ***:**.*::::**** **:**:::**:****:***:*  ** *** **

FiNrdA RYIDQSQSLNIMINPATTTAKEMNQLYLYAWESGIKSLYYQHSTSAAQAFYRSSVCLACE 566
LbNrdA NHIDQGQSINLLVHPDM-PIKEINKIHITAWKLGLKSLYYQHSMNAAQKFKQKKECVSCE 599
       .:***.**:*::::*     **:*:::: **: *:******** .*** * :.. *::**

FiNrdA A 567
LbNrdA A 600
       *
```

Figure S5. BLAST alignment of NrdA sequences from *F. ignava* and *L. blandensis*. The sequence identity is 61%. The alignment was made using Clustal O at the UniProt server (2).

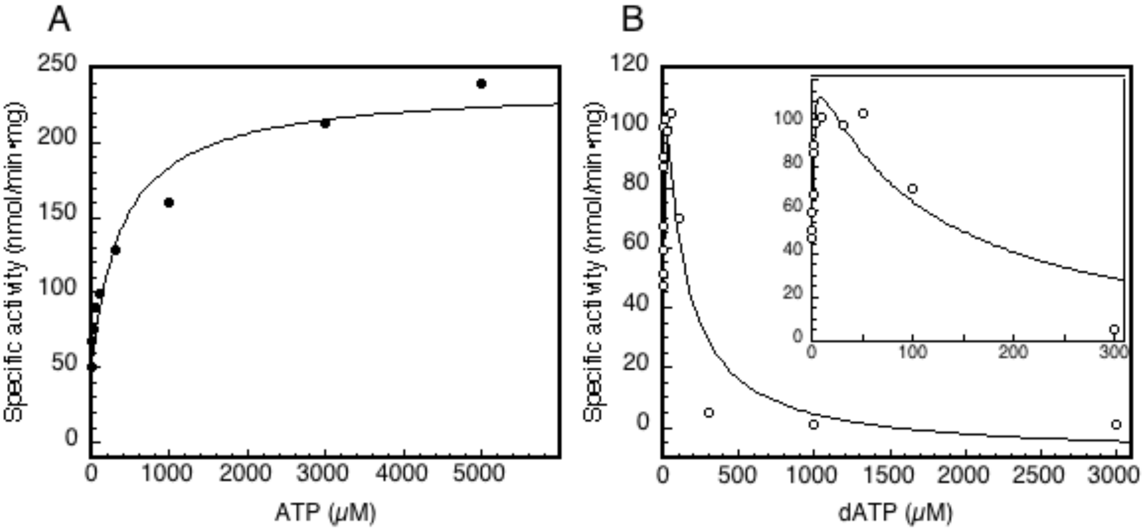

Figure S6. Enzyme activity in heterologous mixtures of *F. ignava* NrdA and *L. blandensis* NrdB. Reduction of CDP as a function of A) ATP concentration giving a K_L of 400 μM, and B) dATP concentration giving a K_i of 100 μM for the ATP-cone in *L. blandensis* NrdB (*Inset*: lower dATP concentrations highlighting the K_L of 1 μM for the specificity site on *F. ignava* NrdA). Two independent binding sites for dATP were evaluated according to the equation: $v=v_{max1}\times[dATP]/(K_{L1}+[dATP])+v_{max2}\times[dATP]/(K_{L2}+[dATP])$.

REFERENCES

1. Rozman Grinberg, I., Lundin, D., Hasan, M., Crona, M., Jonna, V. R., Loderer, C., Sahlin, M., Markova, N., Borovok, I., Berggren, G., Hofer, A., Logan, D. T., and Sjöberg, B. M. (2018) Novel ATP-cone-driven allosteric regulation of ribonucleotide reductase via the radical-generating subunit. *Elife* **7**, e31529
2. The UniProt, C. (2017) UniProt: the universal protein knowledgebase. *Nucleic Acids Res* **45**, D158-D169
